# Editorial: Insights in nanotoxicology

**DOI:** 10.3389/ftox.2026.1960533

**Published:** 2026-08-18

**Authors:** Luisa Campagnolo, Bengt Fadeel

**Affiliations:** 1 Department of Biomedicine and Prevention, University of Rome Tor Vergata, Rome, Italy; 2 Institute of Environmental Medicine (IMM), Karolinska Institutet, Stockholm, Sweden

**Keywords:** artificial intelligence, engineered nanomaterials, nanotoxicology, public dialogue, test guidelines

Nanotoxicology is a relatively new discipline that emerged in response to the rapid growth of nanotechnology and the recognition that materials at the nanoscale may display behaviours in biological and environmental systems that differ from the bulk counterpart (Fadeel, 2019). The field of nanotoxicology has continuously evolved over the past two decades, drawing on a wealth of knowledge in classical particle toxicology (Riediker et al., 2019), and efforts have been made to ensure that more realistic toxicological data are generated to support risk assessment. The six contributions in this Research Topic reflect the broad and evolving landscape of nanotoxicology, and while some articles address general topics and others discuss specific examples, they all touch on the key issue of data interpretation and reliability in nanotoxicology.

Fadeel and Sayre open the discussion with a “conversation” on the lessons learned with respect to the safety assessment of nanomaterials, touching on several of the key issues with which toxicologists have grappled over the past two decades (Maynard et al., 2006). They address the importance of generating OECD-compliant toxicological data using validated protocols or test guidelines to support the regulation and commercialization of new materials and products.

Kühnel et al. discuss science communication, using the projects DaNa, DaNa2.0, and DaNa4.0 (there was no DaNa3.0) as examples. These projects, funded by the German Federal Ministry of Education and Research (BMBF), were designed to develop a trusted repository of data on the safety assessment of nanomaterials to promote public dialogue. The authors concluded that science communication is not a secondary activity performed after the research has been completed but should be seen as an essential part of the research process itself.

Campagnolo et al. also address the question of data quality, especially as this pertains to *in vitro* nanotoxicology, and consider how artificial intelligence (AI) may contribute to the future development of the field. The authors discuss machine-learning approaches to identify relationships between material properties and biological effects, support predictive modelling and promote safe-by-design strategies. However, they emphasise that the usefulness of AI depends on the quality and accessibility of the data since AI cannot compensate for the lack of data.

Graphene is a two-dimensional material with many intriguing properties, but to take advantage of these properties, well-defined and well-characterized materials are needed (Kostarelos and Novoselov, 2014). Andraos et al. critically examine the contradictory toxicological data reported for graphene, graphene oxide and reduced graphene oxide, and show that inconsistencies may arise not only from differences in material intrinsic properties, but also from the interference of graphene and its congeners with commonly used cell viability assays.

The European Food Safety Authority (EFSA) recently concluded, in a reversal of their previous position, that a concern for genotoxicity of titanium dioxide (also known as E171) as a food additive could not be ruled out. E171 was subsequently banned in the European Union, thereby prohibiting any food products containing E171 from being produced or placed on the market. Warheit critically examines the underlying evidence and discusses differences in opinion among other national health-oriented agencies regarding the safety of titanium dioxide.

In the final installment, Kurillová et al. performed an experimental study to address the cytotoxicity of metal nanoparticles. Importantly, by synthesising silver, copper and gold nanoparticles using similar protocols to control for size, shape and surface charge, the authors were able to isolate chemical composition as a major determinant of cytotoxicity (using a mouse embryonic fibroblast cell line as a model). The authors did not specifically address the role of dissolved ions although previous studies have shown that silver and copper nanoparticles can be internalized by cells in particulate form, only to dissolve inside the cell, a mechanism known as the “Trojan horse” mechanism (Valsami-Jones and Lynch, 2015). Nevertheless, the merit of the study rests on the isolation of chemical identity as a driver of toxicity.

Taken together, these contributions provide a snapshot of the scope and evolution of nanotoxicology, highlighting that further progress in this field is dependent not only on generating more data for more nanomaterials, but on producing robust data that can be used to support regulatory decision making. These challenges need to be met in the years to come to ensure that innovation proceeds in tandem with the protection of human health and the environment.
